# Infection rate and factors affecting close contacts of COVID‐19 cases: A systematic review

**DOI:** 10.1111/jebm.12508

**Published:** 2022-12-13

**Authors:** Yunxuan Li, Jing Tan, Suoyi Tan, Yilong Zhou, Bin Sai, Bitao Dai, Xin Lu

**Affiliations:** ^1^ College of Systems Engineering National University of Defense Technology Changsha China; ^2^ Chinese Evidence‐Based Medicine Center National Clinical Research Center for Geriatrics West China Hospital Sichuan University Chengdu China; ^3^ Department of Global Public Health Karolinska Institute Stockholm Sweden

**Keywords:** close contacts, COVID‐19, infection rate, meta‐analysis, SARS‐CoV‐2

## Abstract

**Objective:**

Contact tracing plays an essential role in mitigating the impact of an epidemic. During the COVID‐19 pandemic, studies of those who have been in close contact with confirmed cases offer critical insights to understand the epidemiological characteristics of SARS‐CoV‐2 better. This study conducts a meta‐analysis of existing studies' infection rates and affecting factors.

**Methods:**

We searched PubMed, Web of Science and CNKI from the inception to April 30 2022 to identify systematic reviews. Two reviewers independently extracted the data and assessed risk of bias. Meta‐analyses were conducted to calculate pooled estimates by using Stata/SE 15.1 software.

**Results:**

There were 47 studies in the meta‐analysis. Among COVID‐19 close contacts, older age (RR = 1.94, 95% CI: 1.70, 2.21), contacts in households (RR = 2.83, 95% CI: 2.20, 3.65), and people in close contact with symptomatic infections (RR = 3.62, 95% CI: 1.88, 6.96) were associated with higher infection rates.

**Conclusion:**

On average, each primary infection corresponded to 5.8 close contacts. Among COVID‐19 close contacts, older age and contacts in households were associated with higher infection rates, and people in close contact with symptomatic infections had three times higher risk of infection compared to people in close contact with asymptomatic infections. In general, there are significantly more studies from China about close contacts, and the infection rate among close contacts was lower compared to other countries.

## 1 INTRODUCTION

Contact tracing has played an important role in epidemic prevention and control, narrowing the spread of the virus and effectively reducing the mortality rate.^1^ Since the outbreak of SARS‐CoV‐2, contact tracing has supported other specific measures such as monitoring, testing, and the strict isolation of close contacts at an early stage.^2^ Close contact indicates a close and dangerous encounter in space and time with an infected person during a period of infectious pathogen transmission.^3^, ^4^ With the outbreak of COVID‐19, close contacts were the main route of SARS‐CoV‐2 transmission.^5^ Close contact with a high‐risk exposure to someone infected with SARS‐CoV‐2 yields more robust statistics for inferring future developments of the COVID‐19 pandemic.^6^ Research shows that high‐quality close contact tracing can efficiently control the spread of COVID‐19^7^, ^8^ while enabling development trend predictions of the pandemic and guiding epidemic prevention and control. Governments and management departments have immediately traced and managed the isolation of people in close contact with people infected with SARS‐CoV‐2, and many close contacts and infections have been traced and detected. Plenty of studies of close contact tracing based on different close contact data sets have been conducted, but there are few studies of infection risk factors of close contacts.^9^ Therefore, it is necessary and urgent to summarize the epidemiological features of COVID‐19 close contact.

SARS‐CoV‐2 transmission and exposure risks depend on many factors, including the route of disease transmission, patient characteristics, and environmental factors.^10^ Studies based on different data sets have found different infection rates for people in close contact with symptomatic versus asymptomatic infections, between different age groups, for close contacts within a household versus outside a household, and a variety of total close contacts. One study compared the infection rates of close contact with asymptomatic versus symptomatic SARS‐CoV‐2 infections and confirmed a statistical difference (more infections for people in close contact with symptomatic infections).^11^ Studies also found that adolescent (≤20) and older (≥60) close contacts had different infection rates than each other and the group of total close contacts (higher infection rate of older close contacts than total close contacts, lower infection rate of close adolescent contacts than total close contacts).^12^, ^13^, ^14^ Moreover, significantly higher infection rates were found for close household contacts compared to other types of close contacts.^15^ Thus, multiple factors affected the infection rates of close contact. This article conducts a meta‐analysis–based systematic review of relevant studies of COVID‐19 close contacts and analyzes the factors that affected the infection rates of close contacts. This article is the first systematic review to study the influencing factors of COVID‐19 infection based on close contacts, which is of great significance in studying the key indicators of the COVID‐19 transmission network.

## 2 MATERIALS AND METHODS

### 2.1 Search strategy and selection criteria

This article follows the preferred reporting items for systematic reviews and meta‐analyses (PRISMA) statement to report (Supplementary material). All included studies were retrieved from open‐source databases and were searched and screened by two independent reviewers. During the research, we comprehensively searched for studies that may correlate with infection rates among close contacts of COVID‐19, and terms such as close contacts, COVID‐19, and contact tracing were used, including mesh terms and keywords to search for eligible research. As the studies on the infection rates of COVID‐19 are mostly based on the general population, we use the keyword “close contacts” to limit the retrieval process such that the searched literature are more related to the purpose of the study. Searched databases included Web of Science and PubMed (see Table 1 for the full list). No language was limited. Given that many countries and regions lack relevant data records due to inadequate medical and health care systems and difficulties implementing contact tracing, and that the data on close contacts in China was significantly more comprehensive owing to the massive governmental effort made with epidemic prevention and control, we also included the Chinese academic database China National Knowledge Infrastructure (CNKI) when searching for literature. All retrieved studies were included in the initial screening process.

**TABLE 1:** Databases, search strategies, and number of studies

Database	Search strategies	Number of studies
Web of Science	Close contacts (title) and COVID‐19 (topic)	101
PubMed	Close contacts (title) and COVID‐19 (all fields)	91
CNKI	Close contacts (topic) and COVID‐19 (topic)	322
CNKI	Close contact tracing (topic)	50
Total		564

### 2.2 Data extraction and criteria

Data screening consists of a prescreening process and a two‐stage screening process. Prescreening excluded studies with restricted full‐text access, that is, studies from unopened documents. In the first stage of screening, the criteria for evaluation are that the research object is consistent, the control variables are reasonable and relevant data are included. In the second stage of screening, a predesigned form was developed to input information such as the title, authors, date, location, main study content, research methods, intervention factors, and data about the close contacts of various population groups, including the number of primary infections, close contacts, and secondary infections. Primary infections were the initial COVID‐19 cases, and secondary infections were people infected through close contact with the primary infections (secondary infections all came from close contact). Data from all studies were collated in the predesigned form after excluding studies that restricted access to their full texts. The studies included a variety of affecting factors and data related to infection through close contact. In order to explore specific factors affecting the infection rates of close contacts, data about the periods and locations of these studies, as well as the specific types of close contacts and secondary infections among them, were also recorded.

In the second stage of screening, studies with lower data quality are excluded. Studies with problems such as data repetition (i.e., the same data were reported in another study), irrelevance to the subject, lack of systematic close contact data (i.e., close contact studies based on a single case or a specific population, such as a group of medical staff, college students, etc.), insufficient data volume (i.e., fewer than 1000 close contacts), and overlapping spatiotemporal close contact data (i.e., multiple studies of close contacts in the same place and during the same time) were eliminated using the predesigned form. The studies of close contacts were subdivided according to the source of the close contact (symptomatic infections and asymptomatic infections) and the characteristics of the people in close contact (e.g., adolescent and older age groups, household contacts). Some studies included close contact data for multiple characteristics.

Although there used to be inconsistent criteria in the early days of the COVID‐19 pandemic, close contacts were soon consistently and commonly defined as people who had had close unprotected contact with confirmed cases, suspected cases, or asymptomatic infected persons starting 2 days before symptoms occurred or before positive nucleic acid testing.^16^, ^17^ The specific definition varies slightly between governments and over different time periods (Table 2). Typical variations include extending the identification time from 2 to 4 days before symptoms occurred or nucleic acid sampling, quantifying the duration of exposure (15 min) or the distance (1 or 2 m) from contact with the infected person, etc. For example, before the emergence of the Delta variation, China used 2 days before the onset of symptoms as the time frame for the contact between close contacts and the cases. However, the time frame was extended to 4 days before the onset of symptoms following the surge in Delta cases due to its higher infectivity.^18^

**TABLE 2:** Definition of COVID‐19 close contacts in different countries

Country	Distance	Time of contact	Duration of exposure
China	Close range	From 2 days before onset of symptoms (before Delta) From 4 days before onset of symptoms (during and following Delta)	
USA	< 6 feet	From 2 days before developed symptoms	>15 min
Singapore	< 2 m	From 2 days before onset of symptoms	>30 min
Qatar	< 2 m	Within 2 weeks of identifying positive case	>15 min
Spain	< 2 m	From 2 days before onset of symptoms	>15 min
Switzerland	< 2 m	Up to 48 h before symptom onset or positive test if asymptomatic	>15 min
Australia		Up to 48 h before symptom onset	Face‐to‐face contact 15 min or in an enclosed space 2 h at least

### 2.3 Meta‐analysis

This article presents a systematic review of factors affecting the infection rates of people in close contact with COVID‐19 cases. In the studies included in the meta‐analyses, the infection rate of close contacts was the ratio of secondary infection among close contacts to the total number of close contacts. Heterogeneity tests were conducted before meta‐analyses. If the result of the heterogeneity test were significant, then the effect size of the study included in the meta‐analysis was significant, and a random‐effects model was used; otherwise, a fixed‐effects model was used. The analyses with control groups focused on close contacts of symptomatic infections and asymptomatic infections, adolescent (≤20) and older (≥60) close contacts, and close household contacts. Rate ratio (RR) was used as the effective value, which indicates the ratio of the infection risk of the experimental group to the infection risk of the control group. Meta‐analyses were conducted in Stata/SE 15.1.

The quality of the evidence was appraised by heterogeneity analyses and subgroup analyses, and subgroup analyses were performed mainly based on the time periods and locations of the studies. Moreover, the quality of the result was appraised by publication bias tests and sensitivity analyses. The publication bias of studies was assessed using Egger's test following the meta‐analyses, and sensitivity analyses were conducted to test if the results were stable.

## 3 RESULTS

### 3.1 Basic characteristics of included studies

**FIGURE 1:**
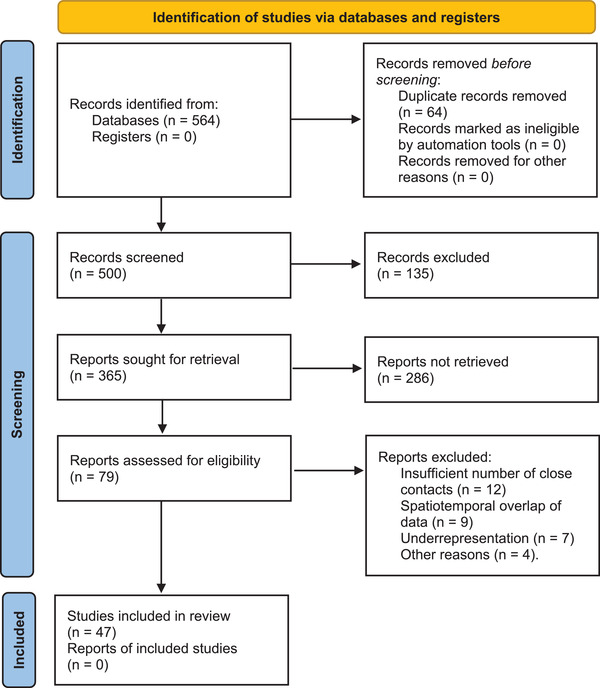
PRISMA flow diagram of the selection process

A total of 564 potential studies were retrieved from 3 databases using 4 search strategies, of which 192 were in English and 372 were in Chinese. The studies span January 1, 2020 to April 30, 2022. The full texts of 500 studies were available for further analysis; 421 studies were excluded due to duplication, topic difference, or lack of systematic data. It is worth emphasizing that studies of single infection cases cannot explain overall infection patterns. Therefore, although plenty of relevant studies of single infection cases were retrieved, they lacked systematic close contact data. The full texts of 79 studies were assessed that contained at least the number of close contacts and secondary infections or the number of primary infections and close contacts. After screening the full texts, 12 studies were excluded because of too few close contact cases, 9 studies were excluded due to spatiotemporal overlap with the close contact data sets of other studies, 7 studies were excluded because they focused solely on specific groups of people and lacked generality, and 4 studies were excluded for other reasons. Finally, a total of 47 studies were included in the meta‐analyses of this article. The detailed selection process is illustrated in Figure 1.

Data on the number of primary infections, close contacts, and secondary infections were extracted from the 47 studies. The common categorizations of the close contact types included household versus other close contacts (23 studies), adolescent and older age versus other close contacts (23 studies), and close contact with symptomatic infections versus asymptomatic infections (9 studies).

### 3.2 Meta‐analyses for binary outcomes

#### 3.2.1 Close contacts of symptomatic infections versus asymptomatic infections

Nine studies contained detailed data on the secondary infection number of close contacts of symptomatic infections and asymptomatic infections.^19^, ^20^, ^21^, ^22^, ^23^, ^24^, ^25^, ^26^, ^27^ The close contact data sets of all 9 studies were from China, indicating that China had more detailed and comprehensive classifications of COVID‐19 close contacts than other countries and focused more on symptoms and the severity of cases.

**FIGURE 2:**
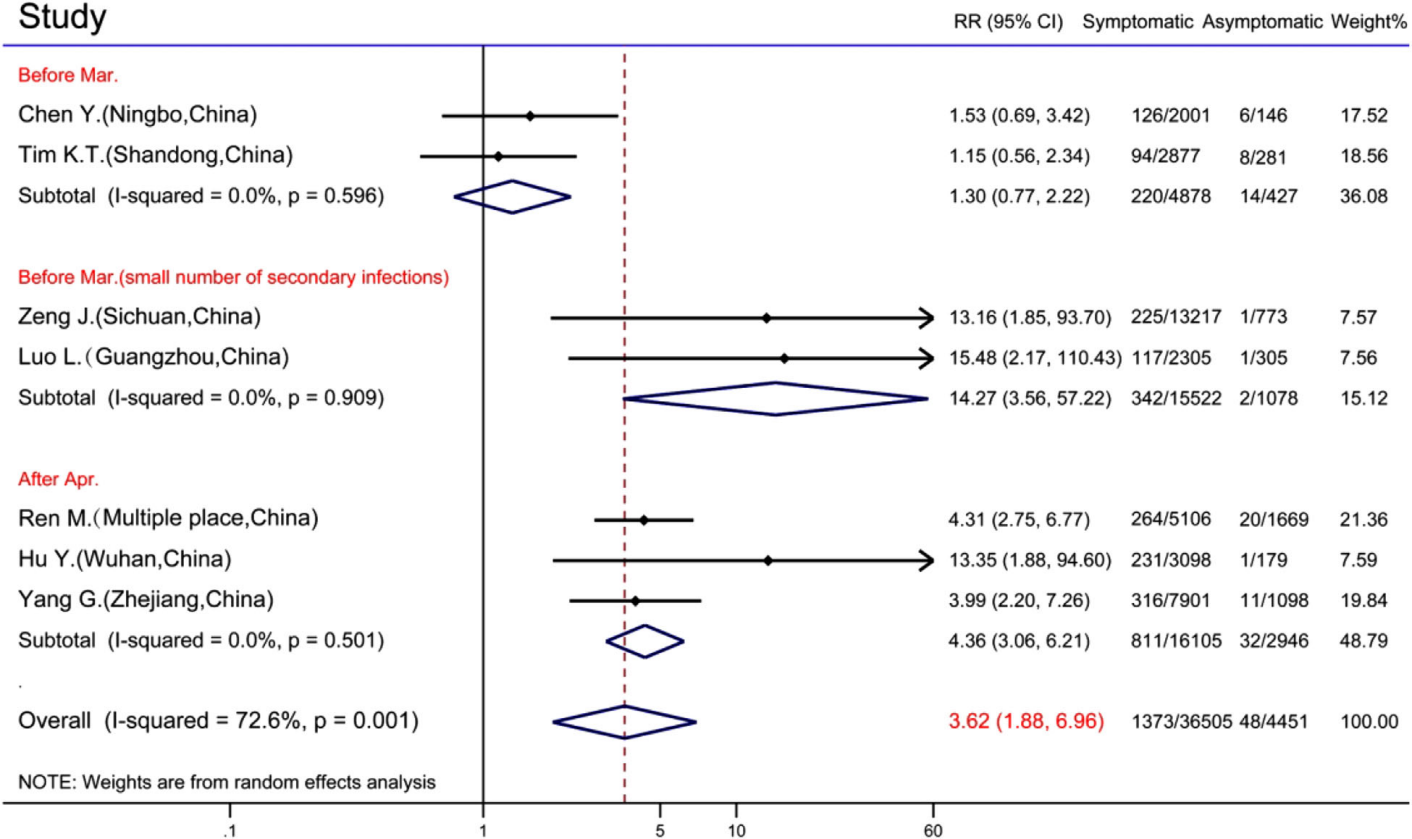
Forest graph of rate ratio (RR) for close contacts of symptomatic and asymptomatic infections

A meta‐analysis for binary outcomes was conducted between close contacts of symptomatic infections and close contacts of asymptomatic infections. Among them, 2 studies were excluded due to lack of rigorous statistics of reported cases^26^ and too small number of close contacts of asymptomatic infections (less than 100).^19^ Close contacts of asymptomatic infections were used as the control group. The average infection rates of close contacts with symptomatic infections versus asymptomatic infections were 3.76% (1 373/3 505) and 1.08% (48/4 451), respectively. The studies showed heterogeneity (*p =* 0.001, *I* ^2^ = 72.6%); therefore, a random‐effect model was used to perform the meta‐analysis. The results showed significant differences in infection rates between close contacts of symptomatic infections and asymptomatic infections, which is consistent with the forest graph shown in Supplementary Figure S1 (RR = 3.62, 95% CI: 1.88, 6.96). The maximum value point estimate for RR at 15.48 (95% CI: 2.17, 110.43) appeared in Guangzhou, China,^24^ and the point estimates of RR were greater than the futility line (RR = 1) in all studies. Due to heterogeneity in meta‐analysis, subgroups were divided according to the cutoff time of the study before or after March 31, 2020, and subgroup analysis was performed. In the first subgroup, studies with a small number of infections in close contacts of asymptomatic infected persons were separately classified into a subgroup because these studies were highly contingent and might cause statistical bias. In the forest graph in Figure 2, homogeneity was reported in each subgroup (*p* > 0.05), indicating that heterogeneity originated from different study times and small sample errors. However, there were no statistical differences among the subgroups.

Meta‐analyses for binary outcomes were conducted between close contacts of symptomatic infections and total close contacts (Supplementary Figure S2) and for close contacts of asymptomatic infections and total close contacts (Supplementary Figure S3). One study was excluded due to a lack of rigorous statistics of reported cases in both two meta‐analyses,^26^ while another study was excluded separately due to too small number of close contacts of asymptomatic infections for the meta‐analysis between close contacts of asymptomatic infections and total close contacts.^19^ The studies of close contacts of symptomatic infections and total close contacts showed homogeneity (*p =* 0.414), while studies of close contacts of asymptomatic infections and total close contacts showed heterogeneity (*p =* 0.006, *I* ^2^ = 66.8%). Therefore, a fixed‐effect model was used for meta‐analysis, which showed that the infection rate of close contacts of symptomatic infections was significantly higher than that of total close contacts (RR = 1.12, 95% CI: 1.04, 1.21). A random‐effect model showed that the infection rate of close contacts of asymptomatic infection was significantly lower than that of total close contacts (RR = 0.32, 95% CI: 0.17, 0.57). A subgroup analysis was performed for the meta‐analysis between close contacts of asymptomatic infections and total close contacts (Supplementary Figure S4). Homogeneity was reported in the subgroup of “After Apr.” (*p =* 0.529), while acceptable heterogeneity was still reported in the subgroup of “Before Mar.” (*p =* 0.008, *I* ^2^ = 74.7%), which caused by the errors of the small number (only one) of close contacts of asymptomatic infections in two studies.^21^, ^24^ The subgroup analysis indicated the heterogeneity originated from different study times. No statistical differences were reported among the subgroups.

#### 3.2.2 Adolescent and older close contacts

**FIGURE 3:**
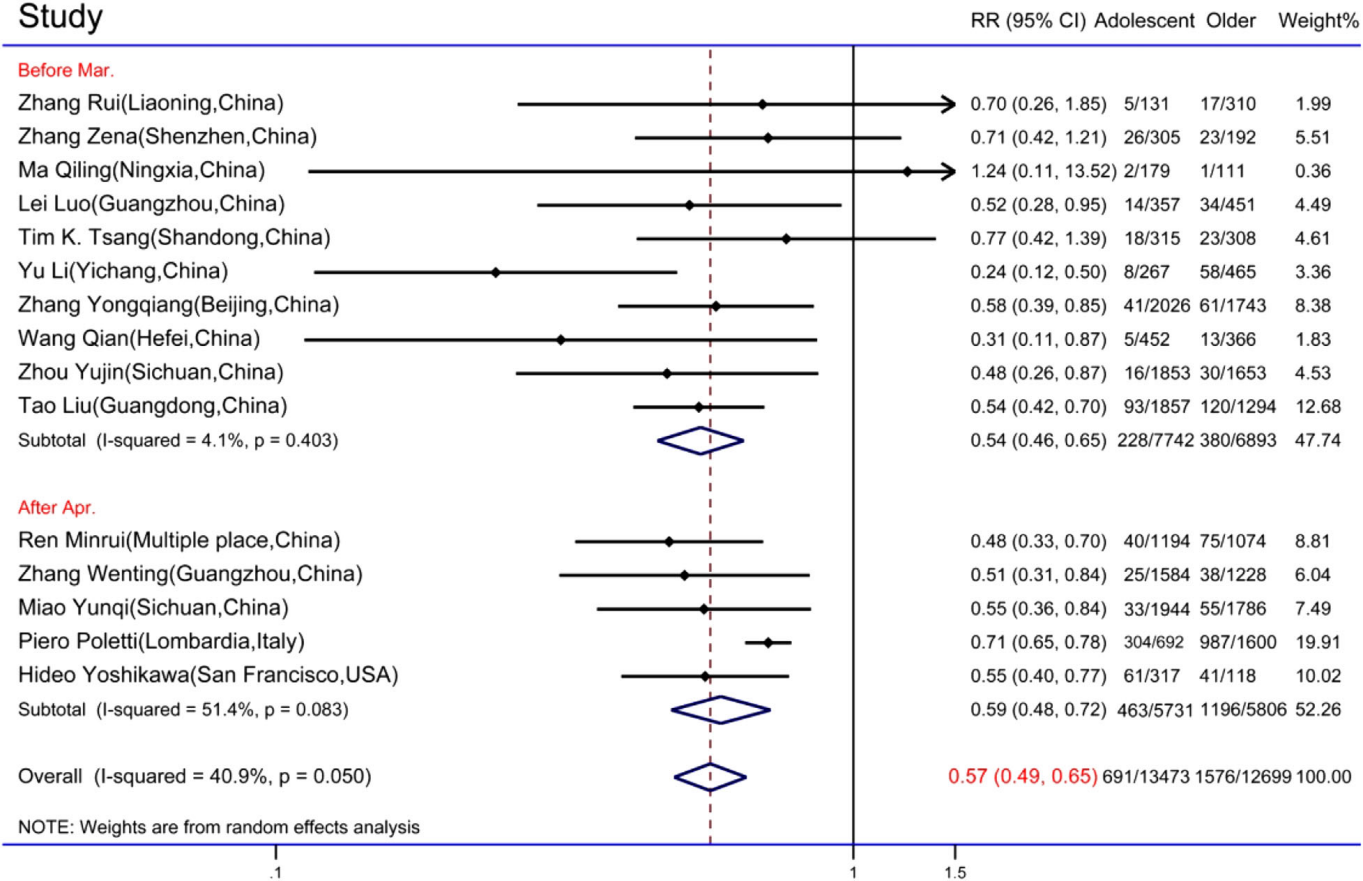
Forest graph of rate ratio (RR) for adolescent and older close contacts

Eighteen studies contained detailed data on the numbers of adolescent and older close contacts.^20^, ^24^, ^25^, ^26^, ^28^, ^29^, ^30^, ^31^, ^33^, ^34^, ^35^, ^36^, ^37^, ^38^, ^39^, ^40^, ^41^, ^42^ The meta‐analysis for binary outcomes used older close contacts as the control group. Three studies were excluded due to a lack of rigorous statistics of reported cases^26^, ^28^ and too small number of older close contacts (less than 100).^37^ Subgroup analysis was shown in Figure 3. Homogeneity was reported in each subgroup (*p =* 0.403 in the subgroup of “Before Mar.” and *p =* 0.083 in the subgroup of “After Apr.”) and total meta‐analysis (*p =* 0.050). The results showed significantly lower infection rates of close adolescent contacts than older close contacts (RR = 0.57, 95% CI: 0.49, 0.65). The point estimates of RR for all studies were below the futility line except for a study in Ningxia, China (RR = 1.24, 95% CI: 0.11, 13.52).

Nineteen studies contained detailed data on adolescent and total close contacts.^20^, ^24^, ^25^, ^26^, ^28^, ^29^, ^30^, ^31^, ^32^, ^33^, ^34^, ^35^, ^36^, ^37^, ^38^, ^39^, ^40^, ^41^, ^42^ The meta‐analysis for binary outcomes used total close contacts as the control group. Three studies were excluded due to a lack of rigorous statistics of reported cases^26^, ^41^ and too small a number of close adolescent contacts (less than 100).^32^ The studies showed heterogeneity (*p =* 0.002, *I* ^2^ = 58.9%), and a random‐effect model was used for meta‐analysis. Subgroup analysis was performed according to the quarter of the study cutoff. Considering the inconsistent definitions of “Adolescent” in different studies, subgroups were divided based on the different definitions of “Adolescent” under 20 years old and under 18 years old (Supplementary Figure S5). After subgroup analysis, homogeneity was reported in each subgroup (*p* > 0.05), indicating that heterogeneity originated from different study times and different definitions of “Adolescent.” No statistical differences were reported among the subgroups. The forest graph reveals that the point estimates of RR, as well as the overall confidence intervals for different studies, were scattered on both sides of the futility line and intersected with it, indicating that there was no significant infection rate difference between adolescent and total close contacts (RR = 1.04, 95% CI: 0.87, 1.24).

Nineteen studies, from China, Iran, Italy, and America, contained detailed data on the number of older and total close contacts.^20^, ^24^, ^25^, ^26^, ^28^, ^29^, ^30^, ^31^, ^33^, ^34^, ^35^, ^36^, ^37^, ^38^, ^39^, ^40^, ^41^, ^42^, ^43^ The meta‐analysis for binary outcomes used total close contacts as the control group. Five studies were excluded due to a lack of rigorous statistics of reported cases^26^, ^28^, ^41^ and too small a number of older close contacts (less than 100).^37^, ^43^ The studies showed significant heterogeneity (*p =* 0.012, *I* ^2^ = 51.9%), and a random‐effect model was used for meta‐analysis. Subgroup analysis was performed according to the quarter of the study cutoff, and homogeneity was reported in each subgroup (*p* > 0.05), indicating that heterogeneity originated from different study times. The infection rate of older close contacts in the second quarter of 2020 (RR = 1.59, 95% CI: 1.33, 1.92) was significantly higher than that of older close contacts in the third quarter (RR = 2.64, 95% CI: 2.15, 3.23). The results showed significantly higher infection rates of older close contacts than total close contacts (RR = 1.94, 95% CI: 1.70, 2.21), as shown in Supplementary Figure S6. All studies' point estimates were above the futility line, and the maximum value point estimate for RR at 2.74 (95% CI: 2.13, 3.54) appeared in San Francisco, USA.^42^

#### 3.2.3 Household close contacts

**FIGURE 4:**
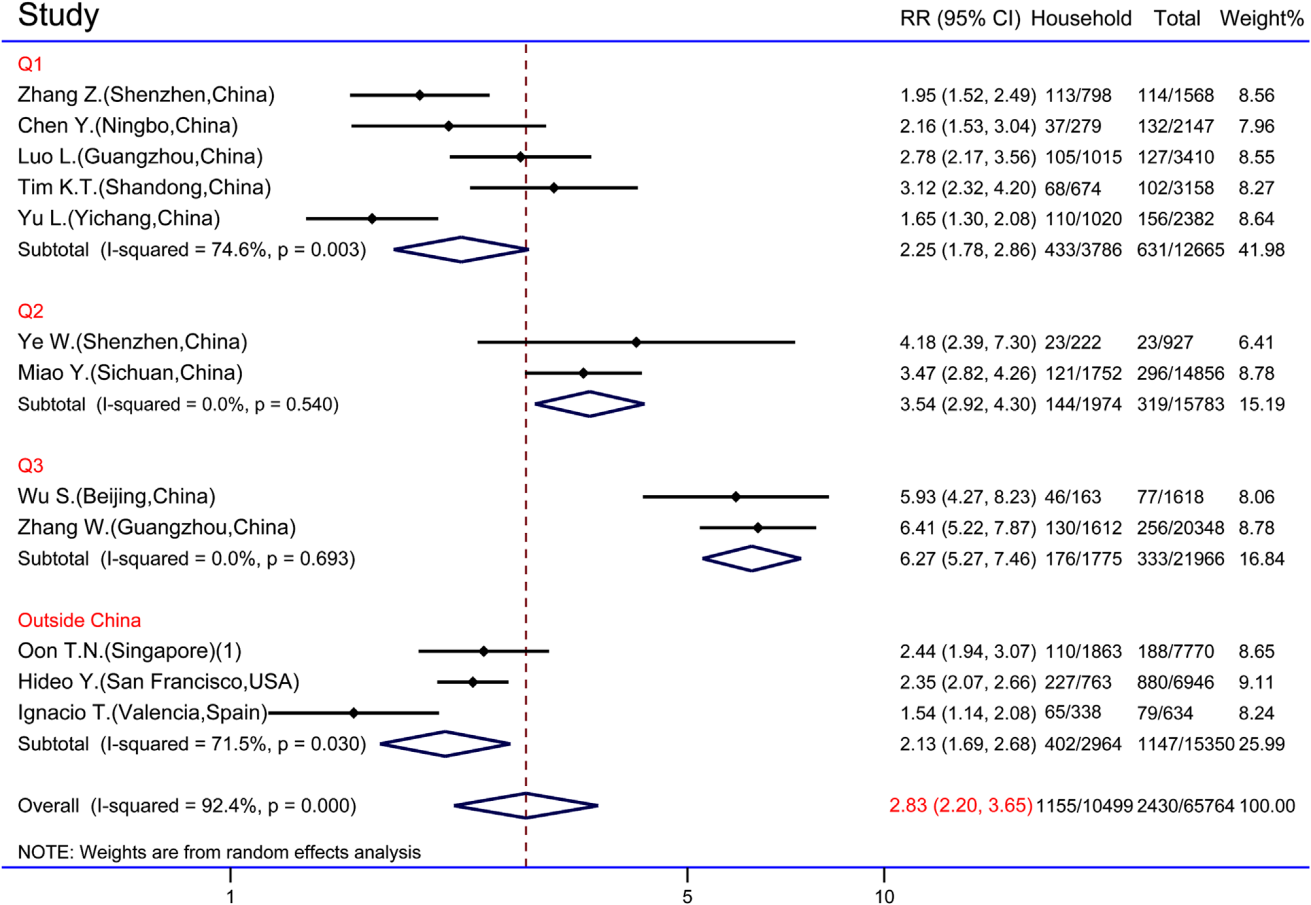
Forest graph of rate ratio (RR) for household and total close contacts

Twenty‐two studies, from China, Iran, Singapore, America, and Spain, contained detailed data on the number of households and total close contacts.^19^, ^22^, ^23^, ^24^, ^25^, ^26^, ^28^, ^29^, ^30^, ^32^, ^33^, ^34^, ^35^, ^36^, ^37^, ^38^, ^39^, ^42^, ^43^, ^44^, ^45^, ^46^ The meta‐analysis for binary outcomes used total close contacts as the control group. We used “live together” as the criterion for identifying close household contacts, and studies declaring close contacts as “family members” were excluded.^19^, ^22^, ^38^, ^43^, ^44^ Moreover, studies that lack rigorous statistics of reported cases were excluded.^26^, ^28^, ^29^, ^35^, ^36^ The studies showed significant heterogeneity (*p <* 0.001, *I* ^2^ = 92.4%), and a random‐effect model was used for meta‐analysis. Subgroup analysis was performed according to the quarter of the study cutoff and the location of the studies. Homogeneity was reported in the subgroups of “Q2” (*p =* 0.540) and “Q3” (*p =* 0.693), while acceptable heterogeneity was still reported in the subgroups of “Q1” (*p =* 0.003, *I* ^2^ = 74.6%) and “Outside China” (*p =* 0.030, *I* ^2^ *=* 71.5%). Heterogeneity originated from different study time and place, and some heterogeneity may cause by statistical errors. In the first three quarters of 2020, there was a statistically significant increase in the infection rate among close household contacts (RR = 2.25, 95% CI: 1.78, 2.86 in 2020 Q1; RR = 3.54, 95% CI: 2.92, 4.30 in 2020 Q2; RR = 6.27, 95% CI: 5.27, 7.46 in 2020 Q3). Results showed significantly higher infection rates of close household contacts compared to total close contacts (RR = 2.83, 95% CI: 2.20, 3.65). The point estimates and confidence interval of RR for all studies were higher than the futility line, and the maximum value point estimate for RR at 6.41 (95% CI: 5.22, 7.87) appeared in Guangzhou, China^34^ (Figure 4).

#### 3.2.4 Publication bias analyses

Egger's test for publication bias was performed on the meta‐analyses for binary outcomes. No significant publication bias was found in the meta‐analyses of close contacts of symptomatic infections and asymptomatic infections (*p =* 0.502), close contacts of symptomatic infections and total close contacts (*p =* 0.367), close contacts of asymptomatic infections and total close contacts (*p =* 0.442), adolescent and total close contacts (*p =* 0.231), older and total close contacts (*p =* 0.549), and household and total close contacts (*p =* 0.712). However, Egger's test showed significant publication bias in the meta‐analysis of adolescent and older close contacts (*p =* 0.010). Therefore, the trim‐and‐fill method^68^, ^69^ was used. However, no study was filled over two iterations (Table 3), indicating no publication bias, and the result of the original meta‐analysis was statistically significant. There was homogeneity after implementing the trim‐and‐fill method (*p =* 0.072, Q = 22.355). To sum up, there was no significant publication bias in this article's meta‐analyses, and the meta‐analyses' results were stable.

**TABLE 3:** Iterations of the trim‐and‐fill method

Iteration	Estimate	Tn	To trim	Diff
1	−0.439	26	0	120
2	−0.439	26	0	18

### 3.3 Systematic review based on study location and study time

Forty‐two studies contained detailed data on the number of close contacts and associated secondary infections;^19^, ^20^, ^21^, ^22^, ^23^, ^24^, ^25^, ^26^, ^27^, ^28^, ^29^, ^30^, ^31^, ^32^, ^33^, ^34^, ^35^, ^36^, ^37^, ^38^, ^39^, ^40^, ^41^, ^42^, ^43^, ^44^, ^45^, ^46^, ^47^, ^48^, ^49^, ^50^, ^51^, ^52^, ^53^, ^54^, ^55^, ^56^, ^57^, ^58^, ^59^, ^60^ therefore, subgroup analyses of the studies' locations and periods were conducted. The study locations were divided into subgroups of China and other countries. Most studies took place in 2020, and studies were divided into the subgroups of Q1 2020, Q2 2020, Q3 2020, and Q4 2020 and later, until the end of the close contact statistical period. Since there was no control group, the point estimations of effect sizes for each subgroup were point estimations of the infection rates for each subgroup.

#### 3.3.1 Location analysis

Twenty‐eight studies occurred in China, while the other 14 were from other countries, including Spain, Iran, Qatar, Singapore, and Mexico. The overall infection rate across 42 studies was 9.3% (95% CI: 7.1%, 11.5%). The infection rate of close contacts in China was significantly lower, which was 3.3% (95% CI: 2.8%, 3.8%). In contrast, the infection rate of close contacts outside China was significantly higher, at 22.0% (95% CI: 13.9, 30.1%), shown in Supplementary Figure S7. Heterogeneity tests showed significance in each subgroup (China: *p <* 0.001, *I* ^2^ = 97.846%; outside China: *p <* 0.001, *I* ^2^ = 99.936%) and among the total studies (*p <* 0.001, *I* ^2^ = 99.912%). Therefore, we expressed the total infection rate of subgroups by summation. The total infection rate of close contacts in China and outside China were 2.73% (4 782/174 989) and 27.04% (37 930/140 257), respectively. The infection rate of close contacts in China was far lower than the close contacts infection outside China, which may result from the strict tracing and isolation policies implemented by the Chinese government.

#### 3.3.2 Time period analysis

The number of studies from Q1 2020, Q2 2020, Q3 2020, and Q4 2020 and later were 17, 9, 11, and 5, respectively. The infection rate of close contacts in Q1 2020 (3.2%, 95% CI: 2.6%, 3.8%) was significantly lower than the overall infection rate, while the other subgroups showed no significant difference compared to the total group. However, the point of estimation of each subgroup showed an increasing trend, which was 9.9% (95% CI: 5.4%, 14.4%) in Q2 2020, 13.3% (95% CI: 8.6, 17.9%) in Q3 2020, and 20.5% (95% CI: 2.6%, 38.4%) in Q4 2020 and later, shown in Supplementary Figure S8. Heterogeneity tests showed significance in each subgroup (Q1 2020: *p <* 0.001, *I* ^2^ *=* 97.431%; Q2 2020: *p* < 0.001, *I* ^2^ *=* 99.853%; Q3 2020: *p <* 0.001, *I* ^2^ *=* 99.800%; Q4 2020 and after: *p <* 0.001, *I* ^2^ *=* 99.987%) and among the total studies (*p <* 0.001, *I* ^2^ *=* 99.912%). Therefore, we expressed the total infection rate of subgroups by summation. The total infection rate of close contacts in Q1 2020, Q2 2020, Q3 2020, and Q4 2020 and later were 2.59% (2 491/96 235), 8.75% (4 416/50 448), 12.56% (7 087/56 440), and 25.61% (28 718/112 123), respectively. The infection rate of close contacts showed an upward trend each quarter of 2020. Studies from China were generally published earlier than those from other countries. Nearly all studies were from China in Q1 2020 and Q2 2020, while studies from other countries were mainly in the other subgroups.

### 3.4 Sensitivity analyses

**FIGURE 5:**
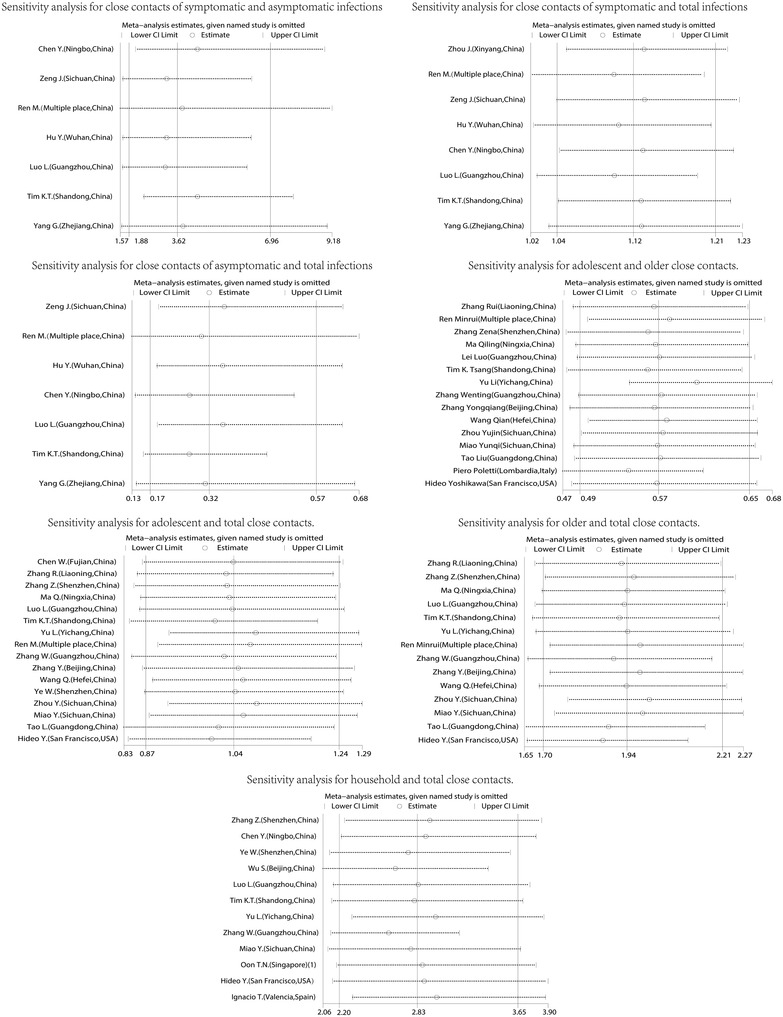
Sensitivity analyses

In order to avoid the influence of low‐quality studies on the results of the meta‐analyses, sensitivity analyses were carried out. In the meta‐analyses, each study was excluded to compare whether the modified meta‐analysis's overall infection rate and confidence interval changed significantly. There was no significant change in the meta‐analyses, indicating the results of these meta‐analyses were stable, as shown in Figure 5.

### 3.5 Analysis of primary infections and the number of close contacts

**FIGURE 6:**
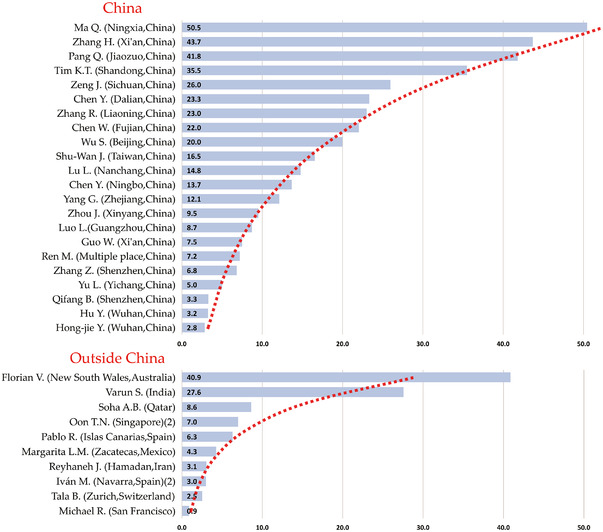
Per capita close contacts of primary cases

Thirty‐two studies contained detailed data on the number of primary infections and close contacts related to them,^19^, ^20^, ^21^, ^22^, ^23^, ^24^, ^25^, ^27^, ^28^, ^29^, ^30^, ^31^, ^32^, ^33^, ^43^, ^44^, ^45^, ^47^, ^48^, ^49^, ^50^, ^51^, ^52^, ^53^, ^54^, ^59^, ^60^, ^61^, ^62^, ^63^, ^64^, ^65^ as shown in Figure 6. On average, each primary case corresponded to 5.8 (36 529/211 972) close contacts. The number of close contacts of confirmed cases per capita in China was 11.2 (7 818/87 838), higher than the number of close contacts of confirmed cases per capita in other countries, which was 4.3 (28 ,711/124 ,134). Per capita close contacts of primary cases essentially followed the Pareto principle. Only four studies in China had more than 30 close contacts per capita, 18.18% of the total number of studies in China. Among the research from other countries, only two studies had more than 20 close contacts per capita, 20% of the total studies outside China, and the remaining studies had less than ten close contacts per capita.

## 4 DISCUSSION

As the group with the highest risk of infection, close contacts are the most critical group to manage for the prevention and controlling of the spread of COVID‐19, and better contact tracing can impede the spread of COVID‐19 more effectively. Analyzing the factors affecting the infection rates of COVID‐19 close contacts has important implications for understanding the transmission of SARS‐CoV‐2. Existing meta‐analyses mainly focus on the infection rate of the total population,^66^ but the infection rates of close contacts lacked analyses.

Existing studies have conducted analyses based on close contact data sets of specific populations to investigate factors affecting the infection rates of close contacts of SARS‐CoV‐2.^19^, ^20^, ^21^, ^22^, ^23^, ^24^, ^25^, ^26^, ^27^, ^28^, ^29^, ^30^, ^31^, ^32^, ^33^, ^34^, ^35^, ^36^, ^37^, ^38^, ^39^, ^40^, ^41^, ^42^, ^43^, ^44^, ^45^, ^46^, ^47^, ^48^, ^49^, ^50^, ^51^, ^52^, ^53^, ^54^, ^55^, ^56^, ^57^, ^58^, ^59^, ^60^, ^61^, ^62^, ^63^, ^64^, ^65^ However, as a new epidemic virus, the epidemiological and clinical characteristics of SARS‐CoV‐2 in the population lack systematic research. The infection rates of people in close contact with COVID‐19 cases may be affected by the source of exposure and age of the contact, among other factors.^23^ Therefore, this article conducted a meta‐analysis of the factors affecting the infection rates of close contacts based on many data sets of close contacts by retrieving relevant studies.

The infection rates of adolescent (≤20) close contacts were not significantly different from overall infection rates, while older (≥60) close contacts had higher infection rates. The infection rates of close adolescent contacts were significantly lower than those of older close contacts. Close contacts in households and close contacts of symptomatic infections were also associated with higher infection rates. Moreover, the infection rates of close contacts gradually increased in 2020.

China reported remarkably lower infection rates among close contacts compared to other countries. However, the per capita close contacts of confirmed cases in China were higher than those in other countries, and the number of per capita close contacts of confirmed cases in each study basically followed the Pareto principle.

It is worth emphasizing that close contacts with symptomatic infections, older close contacts, and close household contacts had significantly higher infection rates. Symptomatic infections may produce more droplets of the virus that are more contagious. Close contacts and infected people in the household environment are in contact for longer periods of time, and the density of virus droplets is higher. Older close contacts are generally less resistant to the virus. This suggests that special attention should be paid to the above types of close contacts in epidemiological investigations and the isolation and medical surveillance of close contacts. These three types of close contacts contain most of the secondary infections and are the key population to control the spread of the epidemic effectively.

A few caveats must be mentioned. First, different definitions of close contacts in the included studies may have somewhat biased the results. Second, several studies needed more specific criteria and detailed descriptions defining close contacts and age subgroups. Moreover, there was significant heterogeneity in single‐proportion meta‐analyses of study time and study location, so total infection rates were used instead of meta‐analyses to calculate the infection risk of close contacts.

Contact tracing technology significantly affects responses to new outbreaks of major infectious diseases. Contact tracing can help identify the characteristics of virus transmission in the first place, block secondary transmission, and identify high‐risk groups. In addition, research on the number and type of close contacts can benefit many related fields. Finally, this article suggests that future studies of close contacts should adopt unified subgroup classification criteria and close contact criteria. As COVID‐19 is still spreading rapidly and the infectious factors of SARS‐CoV‐2 may change as the virus mutates, regular and larger systematic reviews of the infection rates of people in close contact with COVID‐19 cases are necessary to understand the patterns of SARS‐CoV‐2 transmission better.

## CONFLICT OF INTEREST

The authors declare no conflict of interest.

## Supporting information

Supporting Information
